# The transferable resistome of biosolids—plasmid sequencing reveals carriage of clinically relevant antibiotic resistance genes

**DOI:** 10.1128/mbio.02068-25

**Published:** 2025-10-17

**Authors:** Kristin Hauschild, Masato Suzuki, Birgit Wolters, Maho Tokuda, Rin Yamazaki, Megumi Masumoto, Ryota Moriuchi, Hideo Dohra, Boyke Bunk, Cathrin Spröer, Masaki Shintani, Kornelia Smalla

**Affiliations:** 1 Julius Kühn-Institute (JKI), Federal Research Centre for Cultivated Plants, Institute for Epidemiology and Pathogen Diagnostics, Braunschweig, Germany; 2 Antimicrobial Resistance Research Center, National Institute of Infectious Diseases, Japan Institute for Health Security, Tokyo, Japan; 3 Faculty of Engineering, Shizuoka University 13058, Shizuoka, Japan; 4 Graduate School of Integrated Science and Technology, Shizuoka University 13058, Shizuoka, Japan; 5 Shizuoka Instrumental Analysis Center, Shizuoka University 13058, Shizuoka, Japan; 6 Research Institute of Green Science and Technology, Shizuoka University 13058, Shizuoka, Japan; 7 Department of Bioinformatics, IT and Databases, Leibniz Institute DSMZ-German Collection of Microorganisms and Cell Cultures GmbH, Braunschweig, Germany; 8 Japan Collection of Microorganisms, RIKEN BioResource Research Center, Ibaraki, Japan; National University of Singapore, Singapore, Singapore

**Keywords:** sewage sludge, antibiotic resistant bacteria, exogenous plasmid capture, transposons, class 1 integrons, IS-elements, resistance phenotype, wastewater treatment plant, One health, antimicrobial resistance

## Abstract

Biosolids, widely used as organic fertilizers due to their high nutrient content, are significant reservoirs for antimicrobial-resistant bacteria (ARB) carrying transferable antimicrobial resistance genes (ARGs). This study investigated the transferability of ARG-containing plasmids of bacteria from biosolids originating from 12 German wastewater treatment plants (WWTPs) of varying sizes. Using exogenous plasmid captures with the recipient strain *Escherichia coli* CV601 *gfp*+, we collected 103 plasmids from 11 WWTPs. Characterization through DNA-based methods, including real-time PCR and Southern blot hybridization, revealed that the highest proportion of transconjugants harbored IncP (57%) and IncN (20%) plasmids. Complete sequencing of representative plasmids identified IncPβ, IncPε, IncQ2, IncN, and IncU plasmids carrying ARGs linked to mobile genetic elements (MGEs), including class 1 integrons, transposons, and IS elements (e.g., Tn*402*, IS*26*, and IS*6100*). These ARG-MGE complexes were integrated into specific plasmid regions, and similar plasmids were found across WWTPs and diverse geographic locations. The results underscore the role of WWTPs as hotspots for horizontal gene transfer, with biosolids serving as reservoirs for multi-resistant bacteria and resistance plasmids. This highlights the urgent need for improved biosolid management strategies to mitigate the release of ARGs and ARB into agricultural environments.

**IMPORTANCE:**

This study emphasizes the critical role of wastewater treatment plants (WWTPs) in facilitating the horizontal transfer of ARGs through biosolids. As biosolids are routinely applied to agricultural soils, their load of clinically relevant ARG content and transferability pose risks to animal and human health through plant-associated bacteria or surface water. By identifying conserved ARG-MGE associations across diverse plasmid types and WWTPs, this work highlights the global and persistent nature of resistance dissemination. These findings underscore the urgent need for sustainable management practices to limit the spread of antimicrobial-resistant bacteria (ARB) and associated ARGs in agricultural ecosystems. Ensuring safe biosolid use will contribute to combating antimicrobial resistance gene connectivity from environmental to human- or animal-associated bacteria globally.

## INTRODUCTION

The spread of antimicrobial resistance (AMR) has become a global concern, jeopardizing the successful treatment of bacterial infections. Infections that were once treatable now show resistance to most antibiotics (1). AMR extends beyond clinical settings, with environments such as wastewater treatment plants (WWTPs) playing a crucial role in the evolution and dissemination of resistant bacteria. Antimicrobials used by humans select for antimicrobial-resistant bacteria (ARB) not only in hospitals but also in communal environments, as the majority of antibiotics administered to humans are used in households (2). The resistomes—defined as the collection of antimicrobial resistance genes (ARGs)—of different environments seem interconnected through numerous potential pathways, such as the food chain (3, 4). WWTPs are significant reservoirs of ARB, ARGs, and mobile genetic elements (MGEs; collectively referred to as the mobilome, including plasmids and integrons). Additionally, WWTPs accumulate micro-pollutants, such as antibiotics, disinfectants, pharmaceuticals, and metal compounds, which are subsequently released together with ARBs and nutrients into the environment (2). To survive in this environment, bacteria adapt by mutating or acquiring genes that confer resistance or enable the degradation of pollutants. The treated sewage sludge resulting from wastewater treatment, defined as biosolids, is the main end product of this process. They undergo either aerobic or anaerobic stabilization, and their microbiome composition, resistome, and mobilome are influenced by both the treatment process and the sources of wastewater influents (5). Biosolids are often contaminated with heavy metals, disinfectants, detergents, organic pollutants, or pharmaceutical residues (5–7), which can alter soil microbiota when applied to agricultural soils such as fertilizers (8–10).

WWTPs treat wastewater from different sources, including municipalities, industries, and hospitals. This leads to the mixing of a wide variety of bacterial species within close proximity (11). Combined with high bacterial density, biofilms, and exposure to diverse pollutants like heavy metals, biocides, and antibiotics, these conditions promote bacterial stress responses, horizontal gene transfer (HGT), and co-selection, facilitating ARG dissemination among bacteria belonging to different species (12–16). Many organic compounds and ARGs persist through the treatment process, and their potential impact on biosolids applied to soils remains poorly understood (17). Biosolids used as organic fertilizers introduce antibiotics, ARB, ARGs, and MGEs into agricultural soils.

A recent study by Wolters et al. (5) found no correlation between WWTP size and abundance of ARGs, MGEs, or micro-pollutants such as antibiotics and heavy metals. Instead, factors like hospital catchments and aerobic digestion significantly influenced the bacterial community composition and pollutant levels. Biosolids from WWTPs associated with hospitals or food industries harbored high concentrations of quaternary ammonium compounds (QACs) and bacteria from high-risk genera, such as *Acinetobacter*, *Enterococcus*, *Pseudomonas*, *Stenotrophomonas*, and *Streptococcus*. Furthermore, in the study by Wolters et al. (5), diverse MGEs, such as IncP, IncG, IncU, IncN, IncW, IncQ, and PromA plasmids, were detected and quantified by qPCR or Southern blot hybridization in biosolid DNA. However, detection and quantification of plasmids did not allow determining the linkage of the accessory ARGs and MGEs on the different types of plasmids detected. Biosolid amendments not only impact soil microbiomes but also the plant microbiomes and resistomes (18, 19) and facilitate ARG exchange with indigenous soil or plant-associated bacteria (20–23), e.g., through conjugative plasmids.

In the present study, biosolids from the 12 WWTPs of different sizes, previously characterized by Wolters et al. (5), were analyzed for conjugative and mobilizable plasmids. This research aimed to explore the types of plasmids, their accessory ARGs, their linkage to other MGEs, and the resistances conferred. By isolating plasmids through bi-parental matings (exogenous plasmid capturing) and analyzing them via real-time PCR and sequencing, we identified major players involved in the dissemination of ARGs and the role of diverse MGEs through biosolids applied to agricultural soils.

## RESULTS

### Exogenous plasmid isolation

To isolate plasmids from biosolid bacteria, 103 transconjugants resistant to tetracycline (TET) or sulfadiazine (SDZ) were obtained through bi-parental filter matings using *E. coli* CV601 *gfp*+ (rifampicin [RIF] and kanamycin [KAN] resistant) as the recipient. Transconjugants were successfully recovered from 11 of the 12 tested WWTPs (51 and 52 transconjugants from TET and SDZ selective media, respectively), with no recovery from WWTP D due to high background levels of bacteria resistant to KAN, RIF, and TET/SDZ (Table 1).

**TABLE 1:** Number of plasmids isolated from respective WWTPs using tetracycline (TET) or sulfadiazine (SDZ) as selective agent

WWTP^*a*^	Size	WWTP treatment	State of biosolid	Features of catchment area	Plasmids isolated on TET^15^ (mg/L)	Plasmids isolated on SDZ^50^ (mg/L)	Total number of isolated plasmids
A^L^	Large	Anaerobic digestion	Dewatered	Hospital	6	10	16
B^M^	Medium	Anaerobic digestion	Liquid	Hospital, food industry	6	4	10
C^L^	Large	Aerobic stabilization	Liquid	Food industry	9	7	16
D^S^	Small	Aerobic stabilization	Liquid		0	0	0
E^M^	Medium	Aerobic stabilization	Liquid		0	4	4
F^M^	Medium	Anaerobic digestion	Dewatered	Hospital	0	1	1
G^S^	Small	Aerobic stabilization	Liquid		2	3	5
H^M^	Medium	Aerobic stabilization	Liquid		9	2	11
I^S^	Small	Aerobic stabilization	Liquid		4	9	13
J^M^	Medium	Aerobic stabilization	Liquid		1	3	4
K^M^	Medium	Aerobic stabilization	Liquid		10	5	15
L^S^	Small	Aerobic stabilization	Liquid	Amino acid production	4	4	8
Total					51	52	103

^*a*^ Due to confidentiality agreement, only selected size ranges (<10,000; <50,000; <100,000; >100,000 inhabitant equivalents [IEs]) are given. WWTP scale is categorized based on catchment size as follows: >50,000 IEs are categorized as “large”; between 10,000 and 50,000 as “medium”; <10,000 as “small.” The superscript letters (L, M, S) denote the classification.

### Characterization of plasmids captured from biosolid bacteria

Of the 103 transconjugants captured, 57% carried IncP plasmids, detected via real-time PCR and PCR/Southern blot hybridization (Table 2; Table S3). Most IncP plasmids were classified as IncPε and originated from WWTPs of varying sizes. These plasmids frequently harbored *intI1*, *qacE*/*qacE*Δ, *sul1*, and *tetA*. The second most frequently captured plasmids affiliated to the IncN plasmids, representing 20% of transconjugants (23 of 103), were also identified in biosolids across all WWTP sizes. Class 1 integron integrase genes (*intI1*) were present in 70 transconjugants, while class 2 integron integrase genes (*intI2*) were found in two. Sulfonamide RGs (*sul1* and *sul2*) were detected in 69 and 21 transconjugants, respectively. Additionally, 68 transconjugants carried *qacE*/*qacE*Δ, indicating co-localization with *intI1* and *sul1* (Table 2; Table S3).

**TABLE 2:** Characterization of isolated plasmids by TaqMan-based real-time PCR and PCR/Southern blot analyses

WWTP^*a*^	Σ	*korB* (IncP)	*trfA* (IncPε)	*rep* (IncN)	*parA*-*parB* (IncHI1)	*rep* (IncU)	*intI1*	*intI2*	*qacE*/ *qacEΔ*	*sul1*	*sul2*	*tetA*	*tetM*
A-hd^L^	16	16	16	0	0	0	16	0	16	16	0	16	0
B-h^M^	10	10	10	0	0	0	10	0	10	10	0	10	2
C^L^	16	12	9	4	0	0	10	0	10	10	5	10	4
E^M^	4	1	0	0	0	3	4	0	4	4	0	0	0
F-hd^M^	1	1	0	0	0	0	1	1	1	1	0	0	1
G^S^	5	2	2	1	0	1	4	1	4	4	0	2	1
H^M^	11	7	5	0	1	0	8	0	7	7	2	5	4
I^S^	13	1	0	6	0	0	3	0	3	3	3	5	0
J^M^	4	1	1	0	0	0	1	0	1	1	0	1	3
K^M^	15	7	5	8	0	0	7	0	6	7	8	12	1
L^S^	8	3	2	4	0	1	6	0	6	6	3	7	0
Total	103	61	47	23	1	5	70	2	68	69	21	52	16

^*a*^ h, hospital in the catchment area of the WWTP; d, dewatered biosolid; superscript letters S, M, and L denote the classification of the WWTPs into small, medium, and large size.

### Complete sequence of the captured plasmids

From a total of 103 transconjugants, 46 were initially selected for plasmid sequencing. However, due to contamination during DNA extraction, high quality plasmid sequences were obtained from only 12 transconjugants and used for further analysis.. These yielded 17 circular DNA sequences (Table 3). Four plasmids (pKHA1, pKHA7, pKHC1, and pKHJ1) from three different WWTPs showed over 99% identity at the nucleotide sequence level. There were eight IncP plasmids, two IncU plasmids, two IncN plasmids, and one IncQ2 plasmid (Table 3). The remaining sequences included replication initiation genes (pKHI41, pKHI43, and pKHI44) or phage-like elements (pKHL32), none of which carried ARGs.

**TABLE 3:** Plasmid features obtained by exogenous plasmid capture in this study

WWTP^*a*^	Plasmid name^*b*^	Isolated on	Plasmid group	Resitogram^*c*^	Size (bp)	ARGs and their associated MGEs^*d*^	Pc type of integron^*f*^	Tns	Accession number
A-hd^L^	**pKHA1**	Tc15	IncPε-I	**ERY, BAC, TET, SDZ, TMP**	58,705	Tn*402*-class 1 integron (*intI1*, *dfrB1*-IS*26-msrE-mphE*-IS*26*-Δ*intI1*-*qacG2-aadA6-qacG2-qacE*Δ*-sul1-orf5*, *tniB*, *tetRA, eamA,* Δ*tniA*)	PcH1 (*intI1*), PcW (Δ*intI1*)	IS*Pa17*	LC846632
	**pKHA7**	SDZ50	IncPε-I	**ERY, BAC, TET, SDZ, TMP**	58,705	Tn*402*-class 1 integron (*intI1*, *dfrB1*-IS*26-msrE-mphE*-IS*26*-Δ*intI1*-*qacG2-aadA6-qacG2-qacE*Δ*-sul1-orf5*, *tniB*, *tetRA, eamA,* Δ*tniA*)	PcH1 (*intI1*), PcW (Δ*intI1*)	IS*Pa17*	Same as pKHA1
C^L^	**pKHC1**	Tc15	IncPε-I	ERY, **BAC, TET, SDZ, TMP**	58,705	Tn*402*-class 1 integron (*intI1*, *dfrB1*-IS*26-msrE-mphE*-IS*26*-Δ*intI1*-*qacG2-aadA6-qacG2-qacE*Δ*-sul1-orf5*, *tniB*, *tetRA, eamA,* Δ*tniA*)	PcH1 (*intI1*), PcW (Δ*intI1*)	IS*Pa17*	LC846633
	**pKHC10**	SDZ50	IncPε-I	BAC**^*e*^**, **SDZ**	49,607	Tn*402*-class 1 integron (*intI1*, *aadA6-qacE*Δ*-sul1-orf5*, *tniB-tniA*)	PcW	IS*Pa17*	LC846634
G^S^	**pKHG4**	SDZ50	IncU	**ERY,** BAC**^*e*^**, **RIF, AMP, CIP, SDZ**	54,220	*qnrS2*, class 1 integron (*intI1*, *aac(6')-Ib-cr5-bla*_OXA-1_-*catB3-arr-3-qacE*Δ*-sul1-orf5*, IS*6100-mphR-mrxA-* *mphA*-IS*26-aph(3')-Ia*-IS*26*)	PcW		LC846635
I^S^	**pKHI41**	Tc15	rep_cluster_312		51,295	No ARGs	–	IS*5*, IS*As1*, IS*26*, IS*4321*(remnant)	LC846637
	**pKHI42**		IncQ2	ERY, **TET**	15,482	*tetAR, msrE, mphE*	–	IS*Aav2*	LC846638
	**pKHI43**		Not classified		15,191	No ARGs	–		LC846639
	**pKHI44**		Not classified		5,414	No ARGs	–		LC846640
	**pKHI11^p^**	Tc15	IncN	**BAC,** RIF, **AMP, TET,** CIP, **SDZ**	56,616	class 1 integron (*intI1*, *aac(6')-Ib-cr5*-*bla*_OXA-1_-*catB3*-*arr-3-qacE*Δ-*sul1-orf5* IS*6100*), IS*Kpn19-qnrS1-*IS*2*-like remnant*-bla*_LAP-2_-IS*10*, Tn*1721*-related element(*tetRA*)	PcH1	Tn*1721*-like	LC846636
J^M^	**pKHJ1**	Tc15	IncPε-I	**ERY, BAC, TET, SDZ, TMP**	58,705	Tn*402*-class 1 integron (*intI1*, *dfrB1*-IS*26-msrE-mphE*-IS*26*-Δ*intI1*-*qacG2-aadA6-qacG2-qacE*Δ*-sul1-orf5*, *tniB*, *tetRA, eamA,* Δ*tniA*)	PcH1 (*intI1*), PcW (Δ*intI1*)	IS*Pa17*	LC846641
K^M^	**pKHK11**	SDZ50	IncPβ-1	**Hg, SM, AMP, SDZ**	59,405	Tn*3*-like element (*merR*, *merTPADE*, *aph(6)-Id-aph(3'')-Ib*, *tnpR*, Δ*tnpA*, IS*1071*), class 1 integron (*intI1, bla*_OXA-2_-*bla*_OXA-2_-*qacE*Δ*-sul1-orf5*)	PcH1		LC846642
	**pKHK15**	SDZ50	IncPε-I	**SDZ**	48,748	Tn*402*-class 1 integron (*intI1-qacE*Δ*-sul1-orf5*, *tniB, tniA*)	PcW		LC846643
L^S^	**pKHL61^n^**	SDZ50	IncU	**ERY,** BAC**^*e*^**, **AMP, TET, SDZ, TMP**	53,672	*qnrS2*, IS*26-aph(3')-Ia*-IS*26*, *mphA-mrxA-mphR*, class 1 integron (*intI1, aac(6')-Ib-cr5-bla*_OXA-1_-*catB3-arr-3-qacE*Δ*-sul1-orf5*)	PcW	IS*26*	LC846645
	**pKHL62^n^**		IncN		50,859	Tn*3*(*bla*_TEM_), *tetAR*, *sul2-aph(3")-Ib-dfrA14-aph(3")-Ib-aph(6)* *-Id*	–	Tn*3*	LC846646
	**pKHL31**	Tc15	IncPε-I	**ERY, BAC, SM,** TET, SDZ, TMP	58,705	Tn*402*-class 1 integron (*intI1*, *dfrB1*-IS*26-msrE-mphE*-IS*26*-Δ*intI1*-*qacG2-aadA6-qacG2-qacE*Δ*-sul1-orf5*, *tniB*, *tetRA, eamA,* Δ*tniA*)	PcH1 (*intI1*), PcW (Δ*intI1*)	IS*Pa17*	LC846644
	**pKHL32**		Phage-like		41,276	No ARGs	–		Not deposited

^*a*^ h, hospital in the catchment area of the WWTP; d, dewatered biosolid; the superscript S, M, and L denote the classification of the WWTPs into small, medium, and large size.

^*b*^ These sequences were determined by PacBio sequencing and Nanopore sequencing with short-read sequencing.

^*c*^ Antibiotics in bold indicate resistant phenotypes; antibiotics in normal font indicate moderate susceptibility.

^*d*^ Underlining indicates that the corresponding drug resistance phenotype could not be detected.

^*e*^ The corresponding resistance gene could not be identified.

^*f*^ "–,” no integron present.

#### IncP plasmids

**Fig 1:**
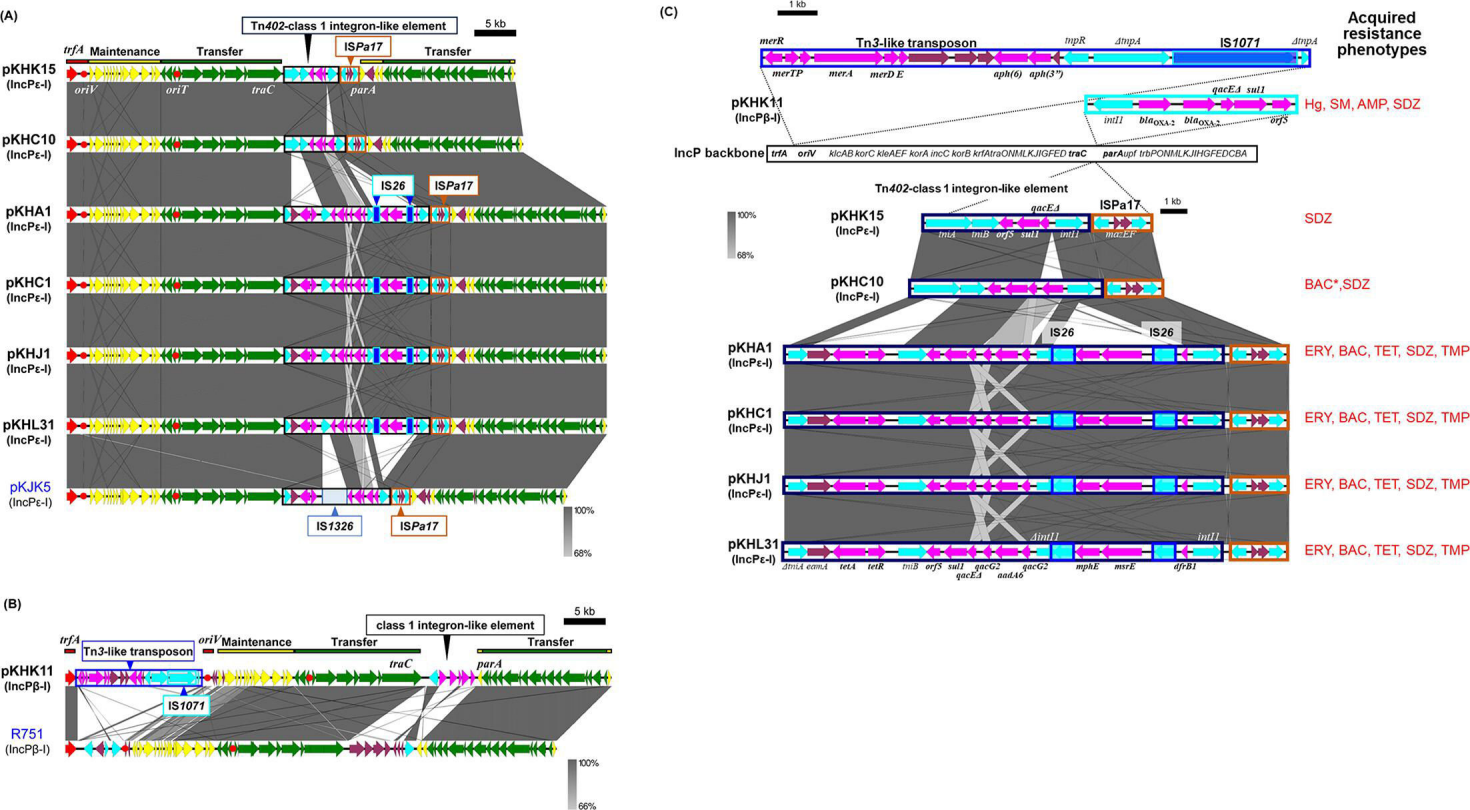
Alignments of IncP plasmids isolated from different WWTPs, IncPε (**A**) and IncPβ (**B**), and those of accessory genes, including antimicrobial resistance genes (ARGs) and other mobile genetic elements (**C**). Coding DNA regions, their directions, and their predicted functions are indicated as block arrows with colors, red for replication, green for conjugation, yellow for other genes in each backbone (shown above or below each plasmid), light blue for genes related to mobile genetic element, pink for genes related to antimicrobial resistance genes, and magenta for other accessory genes. Antimicrobial resistance phenotypes of transconjugants harboring the plasmid are presented using abbreviations for each antimicrobial. An asterisk denotes the absence of corresponding antimicrobial resistance genes (ARGs) in the plasmid sequences. Ampicillin (AMP), benzalkonium chloride (BAC), cefotaxime (CEF), chloramphenicol (CHL), ciprofloxacin (CIP), erythromycin (ERY), gentamicin (GM), mercuric chloride (Hg), meropenem (MER), rifampicin (RIF), sulfadiazine (SDZ), streptomycin (SM), sulfamethoxazole/trimethoprim (SMX/TMP), tetracycline (TET), and trimethoprim (TMP).

Based on phylogenetic analyses of *trfA* and *traI*, seven plasmids (pKHA1, pKHA7, pKHC1, pKHC10, pKHJ1, pKHK15, and pKHL31) were predicted to be IncPε-I plasmids, whereas pKHK11 was assigned as an IncPβ-1 plasmid (Fig. S1). A comparison of the backbones of IncPε-I and IncPβ-1 plasmids with their respective archetype plasmids, pKJK5 (24) and R751 (25), showed that their core genes for replication, maintenance, and transfer were highly conserved (red, yellow, and green arrows in Fig. 1A and B). The accessory genes, including ARGs, were found in their “hot spots,” between *trfA* and *oriV* regions and *traC* and *parA* regions (Fig. 1A through C). The nucleotide sequences of pKHA1 and pKHA7 were identical (58,705 bp), whereas those of pKHA1, pKHC1, pKHJ1, and pKHL31 were slightly different from one another, with 58,701/58,705 nt identical; only one to five bases were replaced. The differences included two synonymous substitutions in different locations in *traL* on pKHJ1, one nucleotide replacement in *mphE* (E-to-K) in pKHA1, and three nucleotide replacements downstream of the recombinase gene in IS*Pa17* in pKHA1.

Tn*402*-class 1 integron-like elements associated with ARGs were found in all seven IncPε-I plasmids (pKHA1, pKHA7, pKHC1, pKHC10, pKHJ1, pKHK15, and pKHL31) and were inserted between *traC* and *parA* (Fig. 1C). Additionally, IS*Pa17* (26) was also found in all of them and contained genes for the MazEF-like toxin-antitoxin system, with inverted repeat sequences similar to those of Tn*402* (Fig. 1C).

Plasmid pKHK15, captured from biosolids of a medium-sized WWTP, contained the archetype Tn*402*-class 1 integron with *qacE*Δ-*sul1*-*orf5*, whereas pKHC10, from a large-sized WWTP, additionally had a streptomycin RG *aadA1* as a cassette gene (Fig. 1C). Resistograms showed that resistance to SDZ was acquired in transconjugants KHK15 and KHC10, and in KHC10, resistance to benzalkonium chloride (BAC) was observed (Table 3). The other five IncPε-I plasmids—pKHA1, pKHA7 (identical plasmid with pKHA1), pKHC1, pKHJ1, and pKHL31—possessed the same Tn*402*-class 1 integrons carrying the trimethoprim (TMP) RG *dfrB1*, two macrolide RGs *msrE* (encoding a ribosomal protection protein) and *mphE* (macrolide phosphotransferase) flanked by IS*26* elements, and another streptomycin (SM)-resistance *aadA6* gene flanked by two copies of multidrug resistance pump genes, *qacG2* and *qacE*Δ-*sul1*-*orf5* (Fig. 1C). These plasmids conferring resistances to multiple antibiotic classes were captured from biosolids of all WWTP sizes. Resistograms revealed resistances to erythromycin (ERY), BAC, TET, SDZ, TMP, sulfamethoxazole/TMP (SMX/TMP) in transconjugants KHA1, KHA7, KHC1, and KHL31. Resistance to SM was only detected in KHL31 (Table 3). All of the IncPε-I plasmids (pKHA1, pKHA7, pKHC1, pKHJ1, and pKHL31) had in common the TET RG, *tetA* inserted into the *tniAB* transposase genes (Fig. 1C).

Regarding pKHK11 (IncPβ-1), a class 1 integron with a Tn*3*-like transposon associated with mercury resistance (*mer*) genes and the aminoglycoside RGs, *aph(6)-Id-aph(3'')-Ib* and IS*1071*, was inserted between *trfA* and *oriV* regions. Another class 1 integron associated with two copies of beta-lactamase gene, *bla*_OXA-2_, was inserted between *traC* and *parA* in pKHK11 (IncPβ-1) (Fig. 1C). Resistograms of transconjugant KHK11 revealed resistances to mercury chloride, SM, ampicillin (AMP), and SDZ (Table 1).

#### IncU plasmids

**Fig 2:**
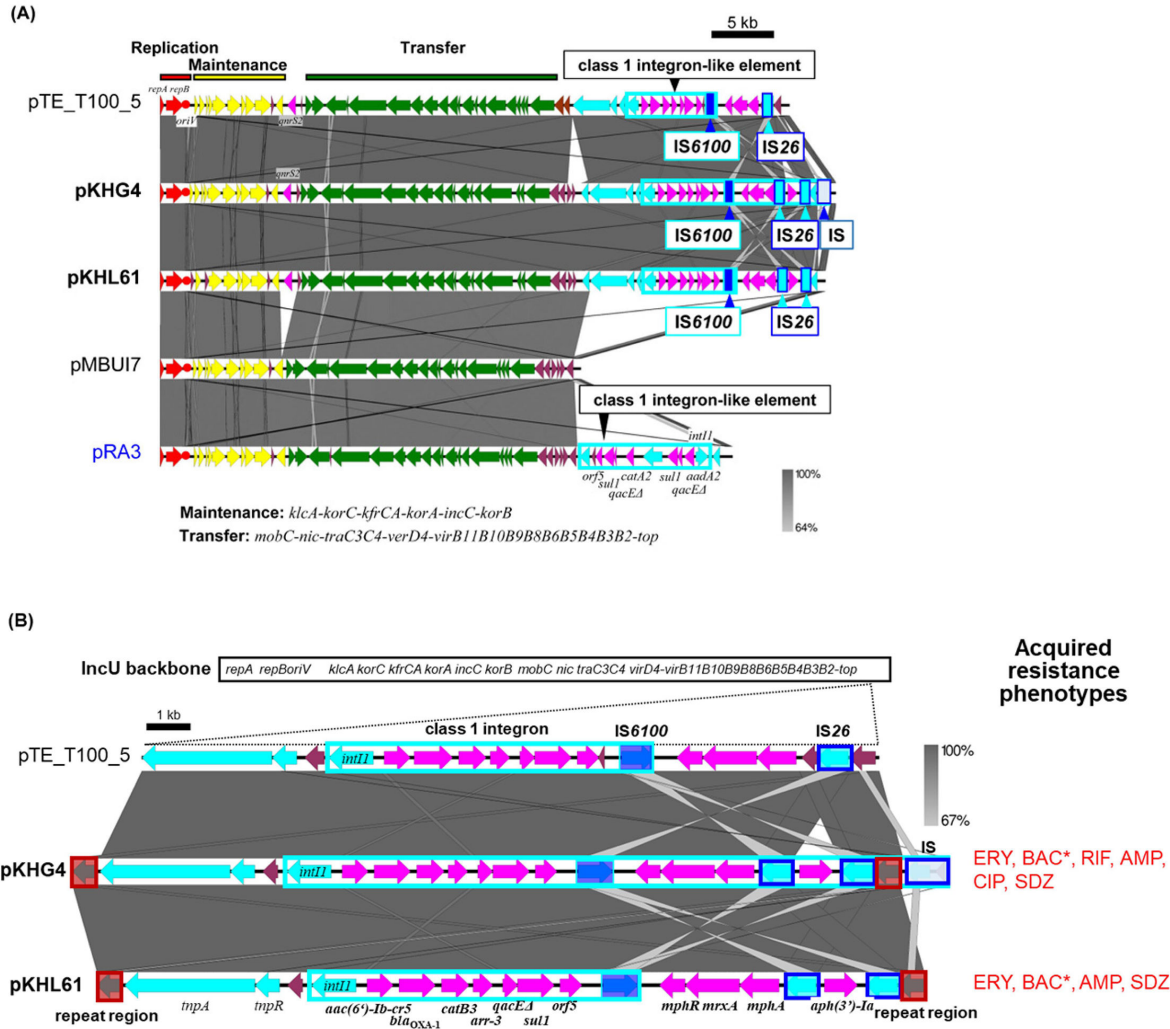
Alignments of IncU plasmids isolated from different WWTPs (**A**) and those of accessory genes including antimicrobial resistance genes (ARGs) and other mobile genetic elements (**B**). Coding DNA regions, their directions, and their predicted functions are indicated as block arrows with colors, red for replication, green for conjugation, yellow for other genes in each backbone (shown above or below each plasmid), light blue for genes related to mobile genetic element, pink for genes related to antimicrobial resistance genes, and magenta for other accessory genes. Antimicrobial resistance phenotypes of transconjugants harboring the plasmid are presented using abbreviations for each antimicrobial. An asterisk denotes the absence of corresponding antimicrobial resistance genes (ARGs) in the plasmid sequences. Parentheses indicate antimicrobials for which ARGs were present but no phenotypic resistance was observed.

Two IncU plasmids (pKHG4 and pKHL61) were isolated from two small WWTPs (G and L) (Table 3). These plasmids shared structural similarity with the IncU archetype plasmid pRA3 (27) (Fig. 2A). Additional comparison with pMBUI7, which was isolated by triparental exogenous plasmid capture from Paradise Creek (Idaho, USA) (28) and contained no accessory genes, indicated that their accessory genes of IncU plasmids were inserted in the upstream region of *repAB* (Fig. 2A and B). The two IncU plasmids pKHG4 and pKHL61 isolated from different WWTPs showed highly conserved region (97.2% identity for 54,714 nt region of both plasmids), and the difference between them was the presence of putative insertion sequence (IS) in the class 1 integron (Fig. 2B). Putative terminal inverted repeats of this IS were similar to those of the terminal region of the class 1 integron, whereas this sequence was not found in the corresponding position in pKHL61. The two plasmids carried the quinolone RG *qnrS2*, the aminoglycoside and ciprofloxacin RG *aac(6')-Ib-cr5*, the beta-lactamase gene *bla*_OXA-1_, the chloramphenicol (CHL) RG *catB3*, and the RIF RG *arr-3*, along with the *qacE*Δ*-sul1-orf5* (Table 3; Fig. 2B). Resistograms of the transconjugants showed that KHG4 transconjugants were resistant to ERY, AMP, ciprofloxacin (CIP), SDZ, and BAC. Rif resistance was conferred by the pKHG4-located *arr-3* gene, after transforming the plasmid into *E. coli* DH5α (as the *E. coli* CV601 *gfp*+ was a RIF-resistant mutant). KHL61 was resistant to ERY, AMP, TET, SDZ, and TMP and moderately susceptible to BAC (Table 3). Interestingly, the two IncU plasmids captured from biosolids were very similar to pTE_T100_5, not only their core genes but also their accessory genes (Fig. 2B). Plasmid pTE_T100_5 was exogenously captured from an effluent sample of municipal wastewater treatment plant in Gothenburg, Sweden (29).

#### IncN plasmids

**Fig 3:**
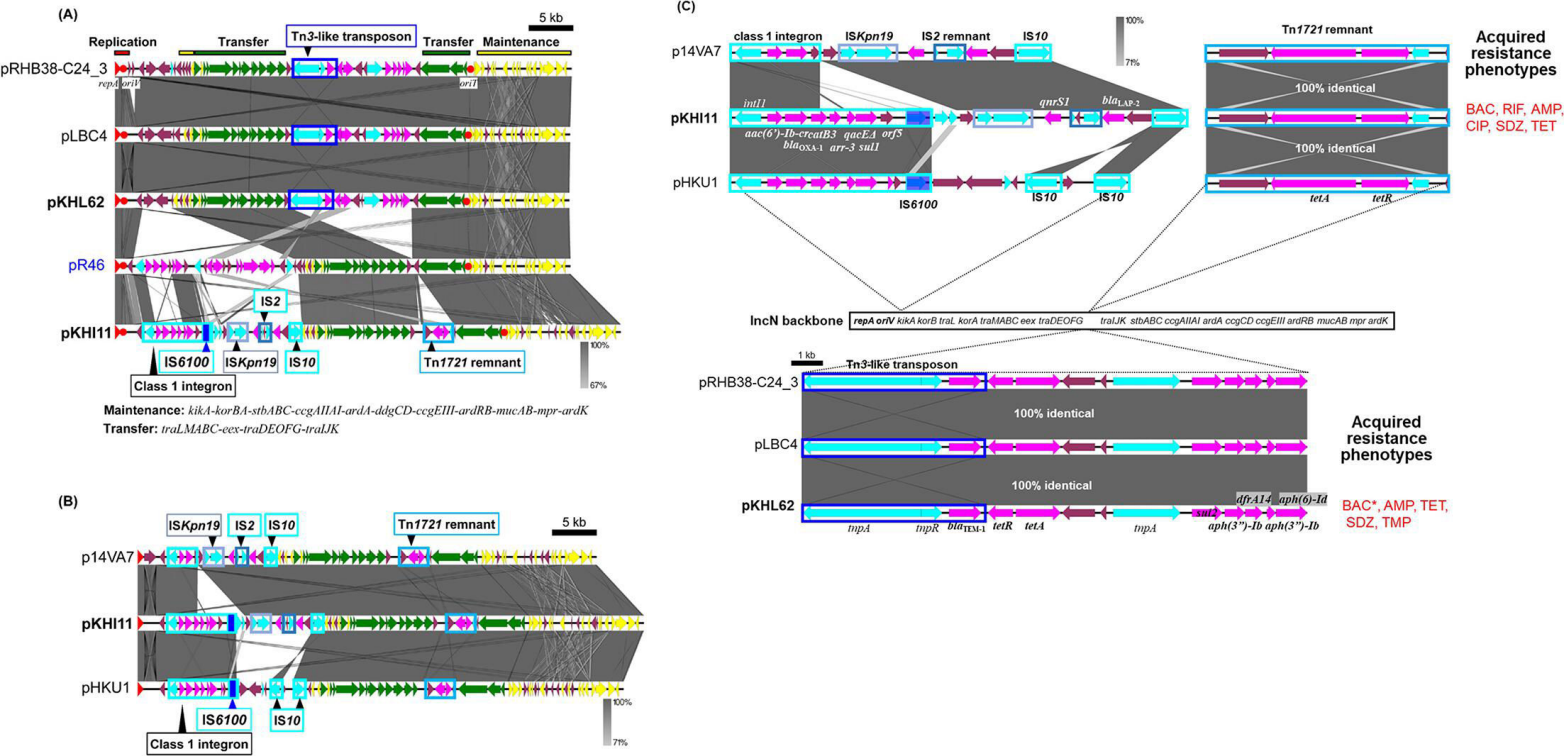
Alignments of IncN plasmids isolated from different WWTPs (**A and B**) and those of accessory genes, including antimicrobial resistance genes (ARGs) and other mobile genetic elements (**C**). Coding DNA regions, their directions, and their predicted functions are indicated as block arrows with colors, red for replication, green for conjugation, yellow for other genes in each backbone (shown above or below each plasmid), light blue for genes related to mobile genetic element, pink for genes related to antimicrobial resistance genes, and magenta for other accessory genes. Antimicrobial resistance phenotypes of transconjugants harboring the plasmid are presented using abbreviations for each antimicrobial. An asterisk denotes the absence of corresponding antimicrobial resistance genes (ARGs) in the plasmid sequences.

The two IncN plasmids (pKHI11 and pKHL62) captured from two small WWTPs (I and L, Table 3) had highly conserved core genes with the IncN1 archetype plasmid pR46 (30, 31)(Fig. 3A). The accessory genes, including ARGs, were inserted either between the replication and transfer region or between the two gene clusters for transfer (Fig. 3A through C). Plasmid pKHI11 possessed a class 1 integron with gene cassettes [*aac*(6')-*Ib-cr5*, *bla*_OXA-1_], along with *catB3*, *arr-3,* and *qacE*Δ-*sul1-orf5* (Fig. 3C). It also carried the fluoroquinolone RG *qnrS1* and the beta-lactamase gene *bla*_LAP-2_, flanked by IS*Kpn19* and IS*10*. Additionally, the TET RG *tetAR* was found in the remnant of Tn*1721* (Tn*3* family) transposon with long inverted repeats (IRs) (245 bp), including the 38 bp IRs conserved in Tn*3*-family transposons, 5 bp direct repeats (DRs), and the functionally inactive transposase gene, Δ*tnpA* (32) (Fig. 3C). Resistograms of transconjugant KHI11 showed that resistances to BAC, AMP, TET, and SDZ, and moderate resistance to CIP and RIF (Table 3). The structure of pKHI11 was similar to those of p14VA7 and pHKU1, both isolated from clinical isolates—*Serratia marcescens* in Japan (accession no. AP028486) and *E. coli* in China, respectively (33) (Fig. 3B and C). Some IS elements were conserved in these three plasmids, flanking the class 1 integron and the Tn*1721* remnants (Fig. 3C). On the other hand, pKHL62 carried a Tn*3*-like transposon with 38 bp IRs containing *bla*_TEM-1_ (Fig. 3C). The pKHL62 additionally carried *sul2*, *aph(6)-Id*, *aph(3'')-Ib*, *tetA*, and *dfrA14* (Table 3; Fig. 3C). Notably, the genetic structure of pKHL62, including their ARGs, was almost identical with those of pLBC4 and pRHB38-C24_3 (Fig. 3C), which were isolated by exogenous plasmid captures from Croatian antibiotic-polluted creek sediment (34) and detected in *E. coli* isolated from pooled pig fecal samples in England (35), respectively.

#### IncQ2 plasmid

**Fig 4:**
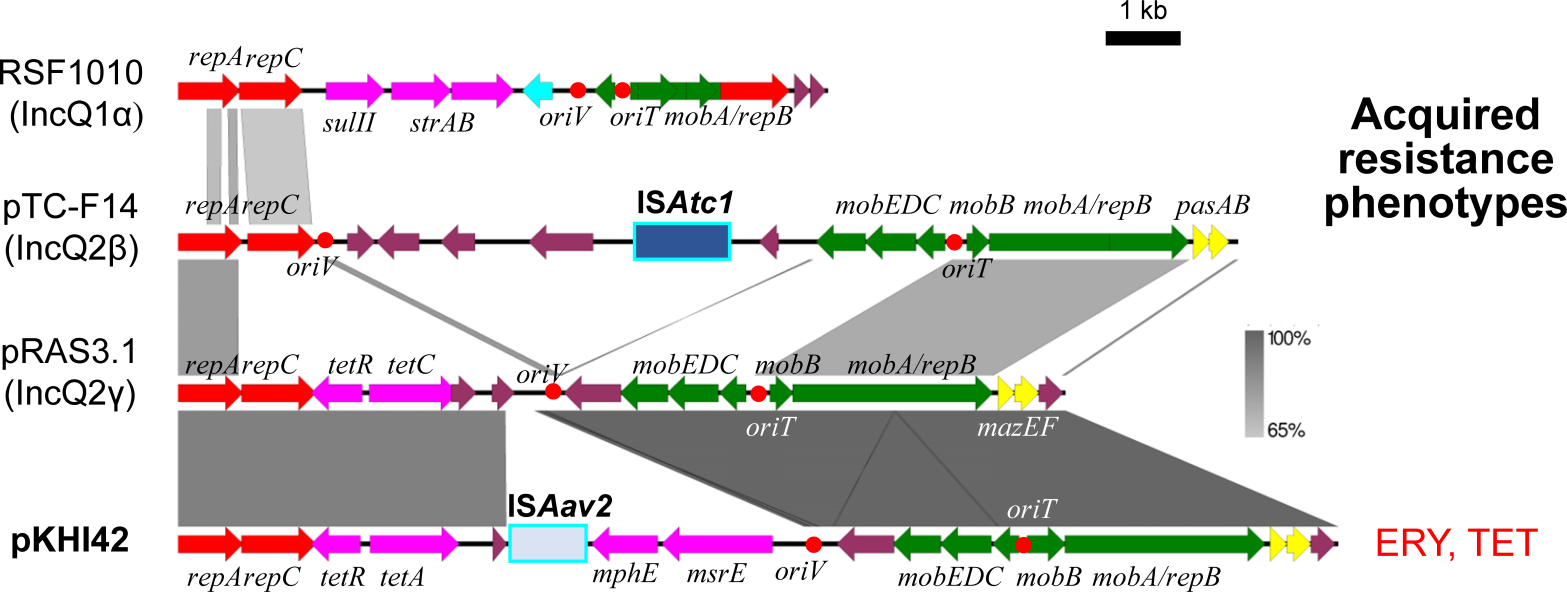
Alignments of IncQ plasmids. Coding DNA regions, their directions, and their predicted functions are indicated as block arrows with colors, red for replication, green for conjugation, yellow for other genes in each backbone (shown above or below each plasmid), light blue for genes related to mobile genetic element, pink for genes related to antimicrobial resistance genes, and magenta for other accessory genes.

Plasmid pKHI42 was assigned to the IncQ2 group, which did not show similarity to the archetype IncQ1 plasmid, RSF1010 (36) (Fig. 4). It had putative replication genes, *mobA/repB* (primase, fusion gene with relaxase), *repA* (helicase), and *repC* (DNA-binding protein), origin of replication (*oriV*), putative mobilization genes, *mobA/repB* (fusion gene with *repB*), and *mobBCDE*, and a toxin-antitoxin (TA)-type plasmid stability system (*mazEF*) similar to the IncQ2 plasmids, pTC-F14 and pRAS3.1, which were found in *Acidithiobacillus caldus*—a sulfur-oxidizing, chemolithotrophic, obligately acidophilic, and moderately thermophilic bacterium isolated from the biooxidation tank used in certain commercial processes (37) and in *Aeromonas salmonicida* isolated from farmed Atlantic salmon (38), respectively (Fig. 4). Plasmid pKHI42 carried *tetA*, *mphE*, *msrE*, and IS*Aav2*, which belongs to the IS*5* family, IS*903* group insertion sequence found in *Paracidovorax avenae* (previous *Acidovorax avenae*) (39, 40), and was inserted between *tetA* and *oriV* regions (Fig. 4). Resistograms of transconjugant pKHI4 revealed resistance to TET and moderate susceptibility to ERY.

Notably, the transconjugant of pKHI42 possessed three more plasmids: pKHI41, pKHI43, and pKHI44 (Table 3). The largest plasmid pKHI41, which was not classified to any of the previously known Inc groups, had *repA* gene identified as rep_cluster_312 by MOB-typer (41), MOB_P_, and MPF_T_ type conjugation system, indicating that it was a self-transmissible plasmid. Like other IncQ2 plasmids, pKHI42 was predicted to be a mobilizable plasmid.

However, it was unclear whether pKHI41, a putative self-transmissible plasmid co-existing in transconjugants of pKHI42, could mobilize pKHI42. Therefore, a filter mating assay was conducted between the transconjugant *E. coli* carrying both pKHI41 and pKHI42 used as the donor and *E. coli* MG1655RG as the recipient. As a result, transconjugants showing TET and gentamicin (GM) resistances were obtained with the frequency around 10^−3^ per donor. Nine transconjugants were isolated and subjected to the genetic analyses, and eight of them possessed both pKHI41 and pKHI42, while the other possessed only pKHI42. Interestingly, no transconjugants had pKHI43 or pKHI44. These results indicated that only IncQ2 plasmid pKHI42 was mobilized by plasmid pKHI41.

#### Class 1 integrons

**Fig 5:**
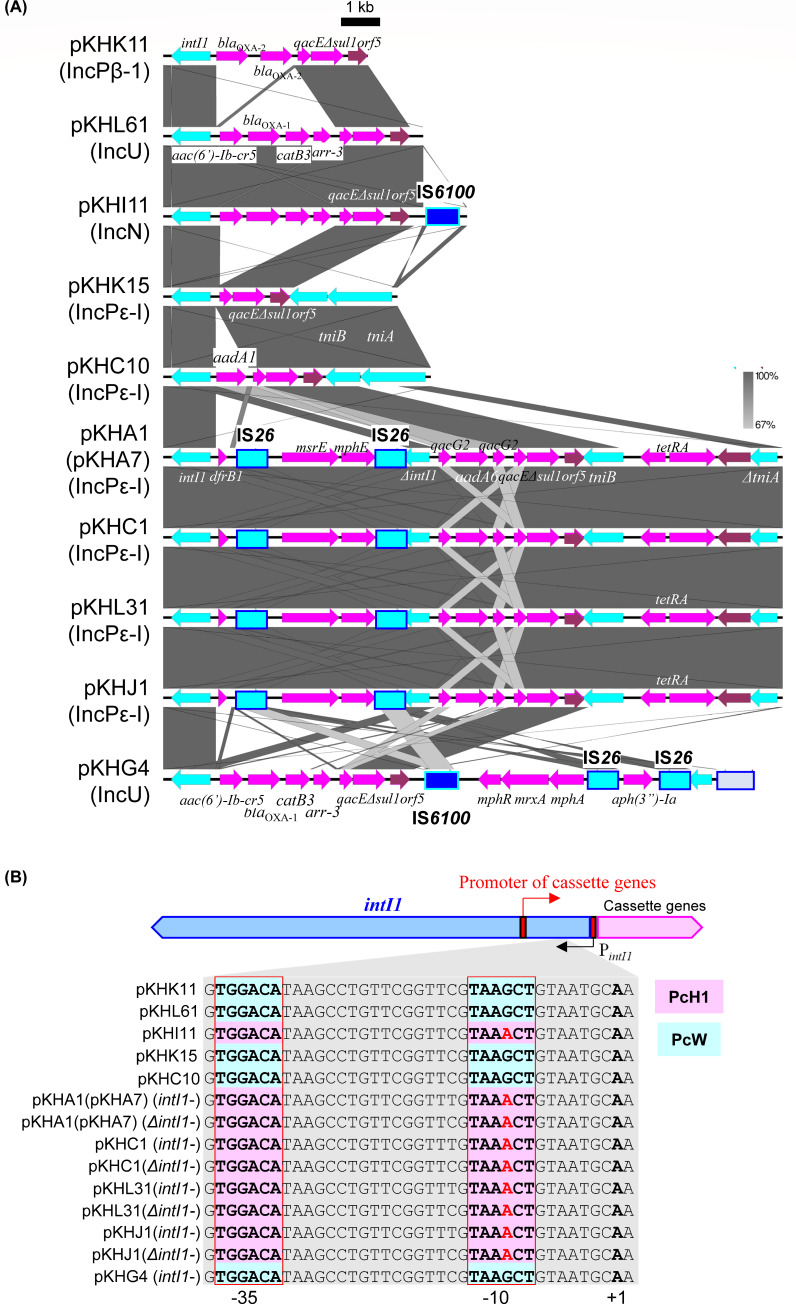
Comparisons of the genetic structure for the class 1 integrons found in different plasmids from different WWTPs (**A**). Coding DNA regions, their directions, and their predicted functions are indicated as block arrows with colors, red for replication, green for conjugation, yellow for other genes in each backbone (shown above or below each plasmid), light blue for genes related to mobile genetic element, pink for genes related to antimicrobial resistance genes, and magenta for other accessory genes. (**B**) Comparisons of promoters for gene cassettes in the class 1 integrons. Red boxes show –35 and –10 regions of the gene cassette and +1 indicates transcription start point. The boxes with light blue indicate PcW promoter, while those with pink indicate PcH1 promoter.

Regardless of the Inc group, class 1 integrons were found in ten plasmids, including pKHA1 (IncPε-I), pKHC1 (IncPε-I), pKHC10 (IncPε-I), pKHG4 (IncU), pKHI11 (IncN), pKHJ1 (IncPε-I), pKHK11 (IncPβ-1), pKHK15 (IncPε-I), pKHL61 (IncU), and pKHL31 (IncPε-I) (Fig. 5A). Plasmid pKHK15 had the simplest Tn*402*-class 1 integron-like element, which had transposase genes *tniA* and *tniB*, and a 3′ conserved segment (3′-CS) with three well-conserved genes: *qacE*Δ*-sul1-orf5* (42) (Fig. 5A; Table 3). The integron of pKHC10 additionally contained the gene cassette *aadA1*, encoding resistance to SM and spectinomycin (Fig. 5A; Table 3). Plasmid pKHK11 had a class 1 integron without *tniAB* genes, containing the beta-lactamase *bla*_OXA-2_ cassette and *qacE*Δ*-sul1-orf5* (Fig. 5A; Table 3). Regarding the integrons of the IncN plasmid pKHI11 and the IncU plasmid pKHL61, the gene cassettes were the same: *aac(6')-Ib-cr5*, beta-lactamase *bla*_OXA-1_, *catB3*, and *arr-3*, with the *qacE*Δ*-sul1-orf5*, whereas the integron of pKHI11 had an IS*6100* insertion in the terminal region (Fig. 5A). Notably, the Tn*402*-class 1 integron-like elements found in the IncPε plasmids pKHA1, pKHA7, pKHC1, pKHL31, and pKHJ1 showed high identity, and only one nucleotide in the integron of pKHA1 was replaced. These IncPε had two *intI1* genes, although one was truncated by the insertion of IS*26* (Δ*intI1*, Fig. 5A; Table 3). The cassette genes of the class 1 integrons were *dfrB1*, *msrE*, and *mphE*, which were flanked by two copies of IS*26* (Fig. 5A). Another cassette gene with Δ*intI1* included the small multidrug resistance efflux pump *qacG2*, *aadA6*, and *qacE*Δ*-sul1-orf5* (Fig. 5A). The *tetRA* was inserted between *tniB* and Δ*tniA* (Fig. 5A). The integron of the IncU plasmid pKHG4 carried the same gene cassettes as those of pKHI11 and pKHL61, with an additional *mphR-mrxA-mphA* operon (Fig. 5A).

Closer comparisons were performed with promoter sequences of cassette genes of integrons, located in the inner region of *intI1* and Δ*intI1*. Each integron of pKHI11, pKHA1, pKHA7, pKHC1, pKHL31, and pKHJ1 had a PcH1 promoter, whereas that of pKHK11, pKHL61, pKHC15, pKHC10, and pKHG4 had a PcW promoter (Fig. 5B) (43).

## DISCUSSION

WWTPs are considered as hotspots for ARB, ARGs, and MGEs (44). While WWTPs are effective in reducing the number of ARB in effluents, biosolids typically still contain micro-pollutants and high numbers of ARB and ARGs (5). The overwhelming majority of studies on ARGs and MGEs in biosolids are currently conducted using DNA-based analysis by means of high-throughput qPCR, amplicon, or shotgun metagenomic sequencing. However, assembly of plasmids from metagenome sequences or whole genome sequences using short reads remains challenging. Thus, the fate of ARGs localized on MGEs, such as plasmids, and their linkage with other ARGs has been largely understudied (45). The detection of ARGs or MGEs in total community DNA provides data on their abundance and diversity, but it does not provide information on the resistance phenotypes, transferability, genetic linkage, or ARG co-localizations. Thus, in the present study, we captured transferable antibiotic resistance plasmids into *E. coli* CV601 *gfp*+ from biosolid bacteria independent of the donor’s cultivability. We showed that transconjugants with transferable plasmids conferring multiple antibiotic resistances were obtained from biosolids independent of the size of the WWTPs. ARG-carrying plasmids from biosolids destined for field application were obtained by exogenous plasmid isolation using either TET or SDZ as a selective agent. We employed these selection markers based on the high concentration of sulfonamides and TETs, as well as the high relative abundance of the corresponding RGs, recently reported by Wolters et al. (5) in the same biosolids studied here. However, due to background growth of donor controls, selecting transconjugants from biosolids was challenging, which explains the low numbers or the absence of transconjugants from WWTP F or D. The high background on donor control plates might be explained by the presence of RIF RG cassette (*arr-3*) identified in the plasmid sequence of the IncU (pKHG4, pKHL61) and IncN (pKHI11) plasmids, indicating transferable RIF resistance in biosolid bacteria. Detailed analyses, following the determination of the full-length nucleotide sequences of several representative plasmids, provided interesting novel insights into the type of plasmids, their genetic relatedness, and acquired RGs linked to various mobile genetic elements.

Therefore, capturing conjugative or mobilizable plasmids into relevant recipient strains allows for testing the captured resistance phenotypes and sequencing of representative plasmids, enabling detailed analysis of co-localized ARGs and their linkage to MGEs, such as class 1 integrons, transposons, and IS elements. In the present study, plasmids were captured from biosolids from 11 of the 12 WWTPs into *E. coli* recipients using TET and SDZ. Notably, the majority of transconjugants carried IncPε plasmids with class 1 integrons, as revealed by real-time PCR, similar to other reported cases where plasmids were collected by exogenous plasmid capture (4, 34, 46, 47). The frequent isolation can be explained by the fact that all conferred resistance to TET of SDZ antibiotics through *tetA* and *sul1*. We selected seven transconjugants with IncP plasmids from WWTPs of different sizes for sequencing to compare their relatedness and the accessory gene load.

### IncP plasmids

IncP group plasmids have a broad host range and are particularly efficient in transferring between bacteria (48–50). They were previously reported to occur in various environments, such as biosolid, wastewater, manure, soils, or river sediments, and are often found in plant-associated bacteria (18, 51–54). The occurrence of IncP plasmids is often associated with pollutants, showing increased abundance in contaminated environments and often carrying RGs for antibiotics, heavy metals, or compounds used in disinfectants (46, 55–57). Notably, many IncPε plasmids, including almost identical IncPε plasmids with class 1 integrons containing the same sets of ARGs and ISs (pKHA1, pKHA7, pKHC1, pKHJ1, and pKHL31, Fig. 1A through C), were isolated from biosolids of WWTPs of varying sizes and anaerobic or aerobic sludge stabilization (Table 1) destined for soil application. The accessory ARGs conferred resistances to the antibiotic pollutants recently determined in the respective biosolids (5). Due to their broad host range and their ability to efficiently transfer in the rhizosphere, these IncP plasmids have a potential to be transferred to human-associated bacteria, possibly via the food chain (1, 18). Additionally, IS*Pa17* with genes for MazEF-like toxin-antitoxin system (26) was commonly found in all IncPε plasmids as well as an archetype plasmid pKJK5 (Fig. 1A and C). Despite the unclear function of plasmid-borne *mazEF* family gene products, their known role in generating stress-adapted translation machinery in *E. coli* (58) implies that this system could aid in the persistence of IncPε plasmids across various environments.

### IncU plasmids

Two plasmids, pKHG4 and pKHL61, both captured from small-size WWTPs that were assigned the archetype of IncU plasmid pRA3 reported in *Aeromonas hydrophila* (59). This plasmid was demonstrated to replicate and be transferred among various bacteria belonging to *Alpha*-, *Beta*-, and *Gammaproteobacteria*, although the transfer frequency to *Alphaproteobacteria* was 1,000-fold lower than those to the others (27). Recently, many IncU plasmids with ARGs have been reported, especially in isolates of the genus *Aeromonas* (60, 61). Comparisons of these plasmids with other different IncU plasmids, including an accessory-gene-free IncU plasmid, pMBUI7, showed that IncU plasmids might have “hot spots” for accessory gene integration—downstream of the putative *oriV* and between genes for conjugative transfer and replication (Fig. 2). It should be noted that class 1 integrons associated with various ARGs were also reported in other IncU plasmids, including pRAS1b found in *A. salmonicida* subsp. *salmonicida* from an Atlantic salmon (62), and pNA6, isolated by bi-parental exogenous plasmid capture from the Haihe River in China (30, 31, 63) (data not shown). These facts suggested that IncU plasmids with class 1 integrons were widely distributed and spread ARGs in different environments.

### IncN plasmids

The archetype IncN plasmid pR46 (R46) was originally found in *Salmonella enterica* subsp. *enterica* serovar Typhimurium (30, 31, 64). The two IncN plasmids, pKHI11 and pKHL62, were captured into *E. coli* from biosolid bacteria of two small-sized WWTPs (Table 1). They possessed conserved backbone regions for replication, maintenance, and conjugative transfer (Fig. 3). Comparison with similar IncN1 plasmids indicated that ARGs were probably inserted at specific sites—either between the replication and transfer region or between the two gene clusters for transfer (Fig. 3). IncN plasmids are frequently found in *Enterobacteriaceae* of human and animal origin (65), conferring resistance against carbapenems and third-generation cephalosporins. However, as recently suggested by Yu et al. (66), this plasmid group is also believed to play a key role in disseminating ARGs in urban water systems.

### IncQ2 plasmid 

Regarding the IncQ plasmid group, four subgroups were suggested: IncQ1–IncQ4 (67). The nucleotide and amino acid sequences of *repA* (helicase) and *repC* (DNA-binding protein) were not highly similar. Thus, real-time PCR and PCR/Southern blot analyses with IncQ (IncQ1) plasmid primers could not assign pKHI42 as an IncQ (IncQ2) group plasmid. pKHI42 showed a genetic structure similar to that of pRAS3.1 found in *Aeromonas* (68) and possessed *tetA* gene, whereas other IncQ2 plasmids usually carried *tetC* (Fig. 4). The transconjugant with pKHI42 had four plasmids (Table 3), with the largest one, pKHI41, predicted to be a self-transmissible plasmid. It had MOB_P_ and MPF_T_ family genes involved in self-transmissibility (Fig. S3). The replicon was assigned as rep_cluster_312 based on MOB typer (41), of which Inc group has not been assigned. This group included p1C73 carrying *bla*_KPC_ (69). Although the pKHI41 did not possess any ARGs, this plasmid could mobilize the IncQ2 plasmid pKHI42.

### Class 1 integrons

The four variants of the gene cassette promoter of class 1 integron located in *intI1* gene (Pc)— PcW, PcH1, PcH2, and PcS—are known to show different transcriptional strengths: PcW (ancestral and the weakest form), PcS (the strongest form), PcH1 (stronger than PcW but weaker than PcH2), and PcH2 (between PcS and PcH1) (43). Two kinds of promoters, PcW and PcH1, of the class 1 integrons were found in the isolated plasmids (Fig. 5). Interestingly, although the cassette genes of the class 1 integrons in pKHL61 and pKHI11 were highly conserved, their promoters differed. The promoter in pKHL61 exhibited lower strength than that in pKHI11 (Fig. 5B). The class 1 integrons with fewer cassette genes, including those in pKHK11, pKHK15, and pKHC10, had the weaker promoter PcW (Fig. 5B). In contrast, those integrons with many cassette genes, including pKHA1, pKHC1, pKHL31, and pKHJ1, had the stronger promoter PcH1 (Fig. 5B). These findings strongly support the notion that increased promoter strength is positively correlated with the development of multidrug resistance.

In several instances, ARGs were identified through molecular screening, yet no corresponding phenotypic resistance was detected (Table 3). Conversely, phenotypic resistance was occasionally observed in the absence of known ARGs. Notably, *catB3* and *aadA6*—genes conferring resistance to CHL and SM, respectively—were detected in several plasmids. However, their corresponding resistance phenotypes were not observed. This may result from insufficient gene expression or impaired functionality of the encoded resistance proteins. In contrast, some transconjugants displayed resistance to BAC despite the absence of known resistance genes, such as *qacG2*. These genes may have been chromosomally integrated, or alternatively, novel resistance genes may exist in these plasmids or the host genome. Notably, these discrepancies derived from sequence data became evident only after performing resistograms for the transconjugants after sequencing.

### Biosolid use and implications

Many WWTPs utilize the high nutritional value of biosolids, a byproduct of the treatment process, as fertilizer for agricultural soils. However, this recycling approach introduces not only nutrients but also pollutants and resistant bacteria into the soil (7, 45). Once in the soil, bacteria from organic fertilizers can exchange genetic material such as ARGs with soil bacteria via HGT, potentially spreading into the food chain through field-grown produce (18, 70). For instance, a study by Law et al. identified six unique plasmids with ARGs in biosolids used as fertilizer, capable of transferring ARGs to human pathogens (53). These plasmids, including three IncPβ, two IncPε, and one PromAβ, have broad host ranges and could persistently spread ARGs between environmental and human-associated bacteria (53).

The corresponding analysis of total community DNA (TC-DNA) isolated from the biosolid (5) revealed a high prevalence of PromA plasmids, as shown by Southern blot hybridization of PCR products obtained from TC-DNA (54, 71, 72). Although PromA plasmids were not found in the captured transconjugants, likely due to their lack of accessory genes for resistance to the selective substances (73, 74) and their poor replication in *E. coli* hosts (75), their presence in biosolid may still contribute to the dissemination of resistance determinants (76).

### WWTPs as hotspots for HGT

Together with the previous report (53), the capturing of resistance plasmids from biosolid and the presence of multi-resistant bacteria confirmed WWTPs’ role as hotspots for HGT. Bacteria carrying transferable resistance genes introduced into agricultural settings via biosolid application might transfer to soil- or plant-associated bacteria and enter the food chain. Drug susceptibility testing results clearly showed that biosolids serve as a reservoir for bacteria equipped with a high diversity of plasmids promoting resistance to multiple antibiotics, some of which are clinically relevant. Additionally, biosolids are often contaminated with potentially selective compounds, such as heavy metals, antibiotics, or disinfectants (5), which can cause selection pressure even at sub-minimum inhibitory concentrations, enhancing HGT processes and increasing the proportion of ARB (77).

### Conclusion

In the present study, we captured conjugative plasmids from biosolid bacteria from differently sized WWTPs that were previously characterized (5) independent of the culturability of their host into *E. coli*. A comprehensive analysis of complete plasmid sequences revealed that clinically significant ARGs, conferring resistance to extended-spectrum β-lactams, macrolide-lincosamide-streptogramin B, fluoroquinolones, or rifampicin, were frequently found co-localized with ARGs conferring resistance to antibiotics commonly used in agriculture, such as sulfonamides or tetracyclines. This co-localization underscores the critical role of co-selection in the spread of antimicrobial resistance. The same or highly similar plasmids were captured from differently sized WWTPs, but comparative sequence analysis also showed the wide geographic dissemination of similar plasmids. Hotspots for the integration of accessory genes or modules seem to be present in the plasmids studied, with transposons, integrons, and IS elements fostering or driving plasmid diversity. We speculate that the plasmids will be beneficial for their hosts, in particular, in the presence of pollutants. Thus, the key to mitigating the dissemination of ARGs is the micro-pollutant reduction during the wastewater treatment.

The overall abundance of ARBs, ARGs, and micro-pollutants is reduced but not entirely eliminated during biosolid treatment, and resistant bacteria have been found in WWTP effluent, indicating incomplete removal (14, 78). In a worst-case scenario, biosolid application on agricultural fields could promote resistance dissemination in produce, the food chain, and the human gastrointestinal tract (18, 19). While biosolid-associated bacteria carrying ARGs and MGEs in soil may decrease over time, some persist at low abundance (57, 79–81) and can proliferate under suitable conditions or be distributed by air (82–84). Overall, in the present study, different broad-host-range plasmids were captured, and their accessory genes mirror the antibiotics and QACs detected in the same biosolids by Wolters et al. (5). The reduction of pollutants and the number of bacteria carrying transferable ARGs released into the environment should be prioritized, as multi-plasmids with broad host range will likely spread under selective conditions and promote ARG connectivity between different environments.

## MATERIALS AND METHODS

### Sampling

Biosolids were collected in spring 2018 from 12 WWTPs in southern Lower Saxony, Germany. Detailed WWTP information is available in Table 1 and Wolters et al. (5). Biosolids were classified as dewatered when water content was reduced using industrial decanters. Four biological replicate samples (250 mL each) were collected from each WWTP, resulting in 48 samples. Samples were stored overnight at 4°C and used the next day for exogenous plasmid isolation.

### Exogenous plasmid isolation

Bi-parental matings were conducted to isolate transferable plasmids, using biosolid-derived bacteria as donors and *E. coli* CV601 *gfp*+ (resistant to kanamycin [KAN] and rifampicin [RIF]) as recipients. Overnight *E. coli* cultures were grown in LB broth with 50 mg/L KAN and RIF at 37°C and 150 rpm. Cultures were centrifuged (3,100 × *g*, 5 min), washed twice in 1/10 tryptic soy broth (TSB), and resuspended in 1 mL 1/10 TSB.

For biosolid preparation, 5 g (solid) or pelleted liquid biosolids per replicate were incubated in 45 mL of 1/10 TSB at 28°C and 150 rpm for 2 h to reactivate donor bacteria. A 1,950 µL aliquot of biosolid suspension was mixed with 50 µL *E. coli* culture, pelleted (3,100 × *g*, 2 min), resuspended in 100 µL of 1/10 TSB, and placed on a sterile 0.22 µm filter (Merck Millipore) on Plate Count Agar (PCA) supplemented with 100 mg/L cycloheximide. Controls included biosolids without recipient and recipient-only suspensions. Filter matings were incubated overnight at 28°C.

The following day, filters were washed in 10 mL of 0.85% NaCl and vortexed for 1 min. Serial dilutions (10⁻²–10⁻⁹) were plated on Mueller-Hinton agar (MH agar) containing 50 mg/L KAN, RIF, 100 mg/L cycloheximide, and either 15 mg/L tetracycline (TET) or 50 mg/L sulfadiazine (SDZ). Control samples were similarly plated. Plates were incubated at 37°C for two days. Transconjugants were confirmed by detection of the *gfp* gene using real-time PCR (85) and BOX-PCR (86).

### Plasmid characterization

Plasmid DNA from transconjugants was analyzed via TaqMan-based real-time PCR to detect sequences associated with plasmid incompatibility groups (IncF, IncI1/I2, IncP, IncPε, IncQ1, and LowGC), class 1 and 2 integron integrase genes (*intI1* and *intI2*), and RGs (*sul1*, *sul2*, *tetA*, *tetM*, and *qacE*/*qacE*Δ). Details of the assays are in Table S1.

Plasmid DNA not assigned to IncP groups was analyzed by conventional PCR and Southern blot hybridization to detect PromA, IncN, IncW, and IncQ sequences (47, 87). Unassigned plasmids were further characterized using the PBRT 2.0 kit (88) (Diatheva S.R.L., Italy).

### Filter mating assay

To confirm mobilization of the IncQ2 plasmid pKHI42 by its helper plasmid pKHI41, filter matings were performed between *E. coli* CV601 *gfp*+ (donor) and *E. coli* MG1655RG (recipient) (54, 72). Selection was based on gentamicin (GM) and TET resistance. Transconjugants were isolated, and plasmid presence was confirmed via PCR using specific primers (Table S1).

### Antimicrobial susceptibility testing for transconjugants of selected plasmids

Susceptibility testing was performed via the disk diffusion method on MH agar following EUCAST guidelines. If no breakpoints for disk diffusion were defined, minimal inhibitory concentrations (MIC) were determined according to EUCAST guidelines. Twelve transconjugant strains and the donor strain were grown on PCA with 50 mg/L KAN and either 15 mg/L TET or 50 mg/L SDZ. For the disk diffusion tests, suspensions (0.5 McFarland turbidity) were prepared, streaked onto MH agar, and tested with antibiotic disks, including ampicillin (AMP), cefotaxime (CEF), chloramphenicol (CHL), ciprofloxacin (CIP), GM, meropenem (MER), SDZ, sulfamethoxazole/trimethoprim (SMX/TMP), TET, and trimethoprim (TMP). Inhibition zones were measured after 24 and 48 h. For MIC testing, liquid cultures were grown in MH broth supplemented with 50 mg/L KAN and either 15 mg/L TET or 50 mg/L SDZ. Suspensions (5 × 10^5^ CFU/mL) were prepared in MH medium supplemented with the following antimicrobial compounds at different concentrations: mercuric chloride (HgCl_2_; 2.5 µg/mL, 5 µg/mL, 10 µg/mL), erythromycin (ERY; 15 µg/mL, 30 µg/mL, 60 µg/mL), streptomycin (SM; 15 µg/mL, 30 µg/mL, 60 µg/mL), and benzalkonium chloride (BAC; 5 µg/mL, 10 µg/mL, 25 µg/mL). Plasmids carrying rifampicin RGs (pKHG4, pKHI11, and pKHL6) were transformed into no-RG-carrying *E. coli* DH5α, and MIC tests with rifampicin (RIF; 25 µg/mL, 50 µg/mL, 100 µg/mL) were performed using transformants instead of transconjugants. MIC testing was performed in 96-well plates for 24 h at 37°C, with OD_600_ measurements taken every 10 min using a modular multimode reader (LB942 TriStar^2^ S, Berthold Technologies, Bad Wildbad, Germany).

### Plasmid DNA sequencing and annotation

Plasmid DNA was sequenced using three different platforms: Illumina MiSeq (short-read sequencing), PacBio RSII (long-read sequencing, Pacific Biosciences), and MinION (Oxford Nanopore Technologies, UK). Libraries were prepared using the Nextera DNA Flex kit (Illumina), SMRTbell libraries (PacBio), or the Ligation Sequencing Kit (ONT), as appropriate. To assemble plasmid sequences from the raw reads, multiple strategies were employed depending on the sequencing platform. For Illumina data, two strategies were applied to avoid chromosomal contamination and assembly artifacts. In strategy i, filtered high-quality MiSeq reads were processed by Trimmomatic v.0.39 (read length > 150 bp and quality score > 15) (89) (301 bp paired-end) and assembled using SPAdes v3.15.3 (90) with the --plasmid option, which is an algorithm for assembling plasmids from whole genome data (91). In strategy ii, only reads that did not align to the host genome (*Escherichia coli* O16:H48 CV601*gfp*, accession NZ_CP043213.1) were assembled to minimize chromosomal sequence contamination. These reads were first mapped using BWA-MEM v0.7.15 (92), and the resulting sam file was converted to the bam file using SAMtools v.1.10 (93). The unmapped reads were then extracted and assembled using SPAdes. The plasmid assemblies from both strategies were identical, supporting the reliability of the results. For long-read data (ONT and PacBio), additional strategies were used. ONT reads were basecalled with Dorado v0.5.3 (https://github.com/nanoporetech/dorado) using the high accuracy (HAC) model and assembled *de novo* using Flye v2.9.3 (94, 95) (strategy iii). The assemblies were then polished twice with Racon (ONT reads) and twice with Pilon (Illumina reads) to improve accuracy. For PacBio data, such as that from pKHI11, assembly was performed using the HGAP3 protocol in SMRTPipe v2.3.0 (strategy iv), followed by circularization and adjustment to the replication initiation gene (*repA*). Repeat sequences and potential chimeric regions were examined using the Repeat Finder plugin (https://www.geneious.com/plugins/repeat-finder) in Geneious Prime 2019.2 (https://www.geneious.com). Final plasmid assemblies were visualized and verified using the Integrative Genomics Viewer (IGV) (96), Qualimap v2.2.1 (97), and Geneious Prime. Gene prediction and annotation were carried out using DFAST-core v1.2.5 (98), followed by manual curation. Full details of all assembly strategies are provided in the Supplemental Text and Table S4.

### Bioinformatical analysis

Plasmid sequences were analyzed and visualized using Easyfig v.2.2.5 (99). *In silico* analyses of insertion sequences, transposons, and genes were performed using Geneious Prime (100). Phylogenetic analyses of replication initiation and relaxase genes were conducted using MEGA7 software (101). Multiple sequence alignments were performed using ClustalW (102), and phylogenetic trees were constructed using the maximum likelihood method with Tamura-Nei and JTT substitution models (103, 104). Annotated plasmid sequences were submitted to DDBJ, with accession numbers listed in Table 3. Accessory genes, including integrons and ARGs, were compared using BLAST. Plasmid maps were visualized using SnapGene and Easyfig. Accession numbers for comparative plasmids are in Table S2.

## Data Availability

The plasmid sequences are available in the DDBJ/GenBank under accession numbers LC846632–LC846646. Their raw sequence data are available under DRA accession number DRA022092 (DRR728437–DRR728450) (https://ddbj.nig.ac.jp/public/ddbj_database/dra/fastq/DRA022/DRA022092/).

## SUPPLEMENTAL MATERIAL

Supplemental Material mbio.02068-25-s0001.pdf Supplemental text and figures.

Supplemental Tables mbio.02068-25-s0002.xlsx Tables S1 to S4.

ASM does not own the copyrights to Supplemental Material that may be linked to, or accessed through, an article. The authors have granted ASM a non-exclusive, world-wide license to publish the Supplemental Material files. Please contact the corresponding author directly for reuse.
