# Supplementary material for: The transferable resistome of biosolids—plasmid sequencing reveals carriage of clinically relevant antibiotic resistance genes
Source: mBio. 2025 Oct 17;16(11):e02068-25. doi: 10.1128/mbio.02068-25 (PMC12607642; doi:10.1128/mbio.02068-25)
Supplement: Supplemental Material — Supplemental text and figures. [file mbio.02068-25-s0001.pdf]

Supplemental materials

The transferable resistome of biosolids – sequencing reveals carriage of clinically relevant antibiotic resistance genes

Kristin Hauschild, Masato Suzuki, Birgit Wolters, Maho Tokuda, Rin Yamazaki, Megumi Masumoto, Ryota Moriuchi, Hideo Dohra, Boyke Bunk, Cathrin Spröer, Masaki Shintani*, and Kornelia Smalla*

Correspondence: shintani.masaki@shizuoka.ac.jp; kornelia.smalla@julius-kuehn.de.

The Supplementary Materials include:

Supplemental Text S1

Figures S1-S3

Tables S1-S4 (provided in Excel format)

Supplemental Text S1

Plasmid extraction and sequencing

Plasmid DNA was extracted from transconjugants using the Plasmid Midi Kit (Qiagen, Germany), following the manufacturer's instructions. The plasmid DNA quantity was assessed by fluorimetry (Qubit 3.0, Thermo Fisher Scientific, USA), while qualitative assessment was performed with the FragmentAnalyzer device (Advanced Analytical, USA). High molecular weight plasmid DNA was sheared with g-TUBE (Covaris, UK) aiming at DNA fragments of about 10 kb. SMRTbell™ library for pKHI11 was prepared according to the instructions from PacificBiosciences, Menlo Park, CA, USA, following the "Procedure & Checklist Preparing SMRTbell Libraries using PacBio Barcodes Adapters for Multiplex SMRT Sequencing". Briefly, for preparation of 10kb libraries 1 to 4µg of each plasmid DNA was sheared using g-tubes™ from Covaris S220, Woburn, MA, USA according to the manufacturer´s instructions. DNA was end-repaired and ligated overnight to barcoded SMRTbell™ adapters applying components from the DNA/Polymerase Binding Kit P6 from Pacific Biosciences, Menlo Park, CA, USA. Reactions were carried out according to the manufacturer´s instructions. Samples were either Exonuclease treated for removal of incompletely formed reaction products or a BluePippin™ Size-Selection to greater than 4 kb was performed according to the manufacturer´s instructions (Sage Science, Beverly, MA, USA). Conditions for annealing of sequencing primers and binding of polymerase to purified SMRTbell™ template were assessed with the Calculator in RS Remote, PacificBiosciences, Menlo Park, CA, USA. SMRT sequencing was carried out on the PacBio *RSII* (PacificBiosciences, Menlo Park, CA, USA) taking one 240-minutes movie on a single SMRT cell. The library for Illumina sequencing (300-bp paired-end for MiSeq) was prepared using the Nextera DNA Flex Licrary Ppreparation Kit (for MiSeq, Illumina) and the library for ONT sequencing was prepared using the Rapid Barcoding Kit (ONT, UK).

Plasmid DNA assembly and annotation.

For plasmid pKHI11, assemblies obtained by multiplexed SMRT sequencing were performed using the HGAP3 Whitelisting protocol within SMRTPipe 2.3.0 applying a genome size of 100 kb and a minimum subread length of 1 kb after demultiplexing using the RS_Subreads.1 protocol contained within SMRT Portal 2.3.0. The circularization was performed by removal of artificial redundancies at the ends of the contigs and adjustment to *repA* as plasmid replication gene [strategies (iv)].

The annotated genes of the IncP/P-1 plasmids were reannotated and named according to those in R751(1), except for *kfrB* (upf54.8, R751) and *kfrC* (upf54.4, R751). The conserved genes in the IncN, IncU, and IncQ2-like plasmids were reannotated and named based on those in R46 (IncN, GenBank accession no. AY046276), pRA3 (IncU, accession no. DQ401103), and pRAS3.1 (IncQ2-like, accession no. AY043298). Accessory genes, including putative metabolic genes,

and/or transporter genes, were subjected to BLAST (https://blast.ncbi.nlm.nih.gov/Blast.cgi) to find similar sequences. Genotypic screening of antibiotic resistance genes in these plasmids was performed by using the Comprehensive Antibiotic Resistance Database (CARD 3.2.5) and Resistance Gene Identifier (RGI 6.0.0) (2, 3).

Supplemental Figure legends

Figure S1. Phylogenetic trees of IncP/P-1 plasmids, inferred from nucleotide sequence data of 40 *trfA* genes (A) and *traI* genes (B), and their amino acid sequence data of TrfA proteins (C) and TraI proteins (D). Plasmids obtained in this study are shown in red. Bootstrap percentages at nodes were calculated with 1000 replicates. The tree is drawn to scale, with branch lengths measured in the number of substitutions per site (indicated by horizontal scale bars). Accession numbers of nucleotide sequences are shown in Table S2.

Figure S2. Phylogenetic trees of IncQ plasmids, inferred from nucleotide sequence data of 15 *repA* genes (A) and *repC* genes (B), and their amino acid sequence data of RepA proteins (C) and Rep proteins (D). Plasmids obtained in this study are shown in red. Bootstrap percentages at nodes were calculated with 1000 replicates. The tree is drawn to scale, with branch lengths measured in the number of substitutions per site (indicated by horizontal scale bars). Accession numbers of nucleotide sequences are shown in Table S2.

Figure S3. Alignments of unclassified plasmids with RIP gene of rep_cluster_312 isolated from different WWTPs. Coding DNA regions, their directions, and their predicted functions are indicated as block arrows with colors, red for replication, green for conjugation, yellow for other genes in each backbone (shown above or below each plasmid), light blue for genes related to mobile genetic element, pink for genes related to antimicrobial resistance genes, and magenta for other accessory genes.

References

1. Thorsted PB, Macartney DP, Akhtar P, Haines AS, Ali N, Davidson P, Stafford T, Pocklington MJ, Pansegrau W, Wilkins BM, Lanka E, Thomas CM. 1998. Complete sequence of the IncPbeta plasmid R751: implications for evolution and organisation of the IncP backbone. J Mol Biol 282:969–990.
2. Alcock BP, Raphenya AR, Lau TTY, Tsang KK, Bouchard M, Edalatmand A, Huynh W, Nguyen A-LV, Cheng AA, Liu S, Min SY, Miroshnichenko A, Tran H-K, Werfalli RE, Nasir JA, Oloni M, Speicher DJ, Florescu A, Singh B, Faltyn M, Hernandez-Koutoucheva A, Sharma AN, Bordeleau E, Pawlowski AC, Zubyk HL, Dooley D, Griffiths E, Maguire F, Winsor GL, Beiko RG, Brinkman FSL, Hsiao WWL, Domselaar GV, McArthur AG. 2020. CARD 2020: antibiotic resistome surveillance with the comprehensive antibiotic resistance database. Nucleic Acids Res 48:D517–D525.
3. Alcock BP, Huynh W, Chalil R, Smith KW, Raphenya AR, Wlodarski MA, Edalatmand A, Petkau A, Syed SA, Tsang KK, Baker SJC, Dave M, McCarthy MC, Mukiri KM, Nasir JA, Golbon B, Imtiaz H, Jiang X, Kaur K, Kwong M, Liang ZC, Niu KC, Shan P, Yang JYJ, Gray KL, Hoad GR, Jia B, Bhando T, Carfrae LA, Farha MA, French S, Gordzevich R, Rachwalski K, Tu MM, Bordeleau E, Dooley D, Griffiths E, Zubyk HL, Brown ED, Maguire F, Beiko RG, Hsiao WWL, Brinkman FSL, Van Domselaar G, McArthur AG. 2023. CARD 2023: expanded curation, support for machine learning, and resistome prediction at the Comprehensive Antibiotic Resistance Database. Nucleic Acids Res 51:D690–D699.

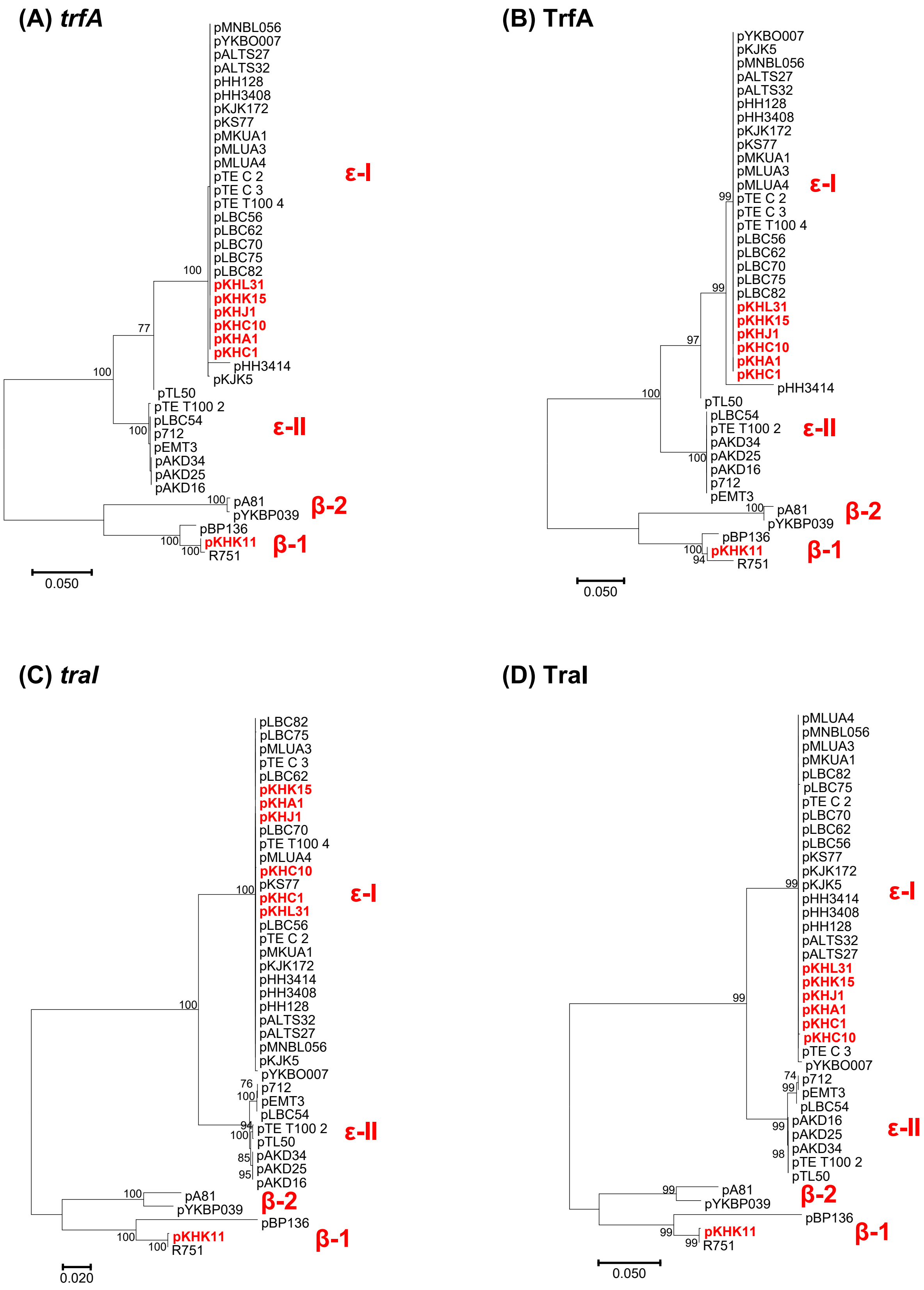

Figure S1. Phylogenetic trees of IncP plasmids, inferred from nucleotide sequence data of 40 *trfA* genes (A) and *traI* genes (B), and their amino acid sequence data of TrfA proteins (C) and TraI proteins (D). Plasmids obtained in this study are shown in red. Bootstrap percentages at nodes were calculated with 1000 replicates. The tree is drawn to scale, with branch lengths measured in the number of substitutions per site (indicated by horizontal scale bars). Accession numbers of nucleotide sequences are shown in TableS2.

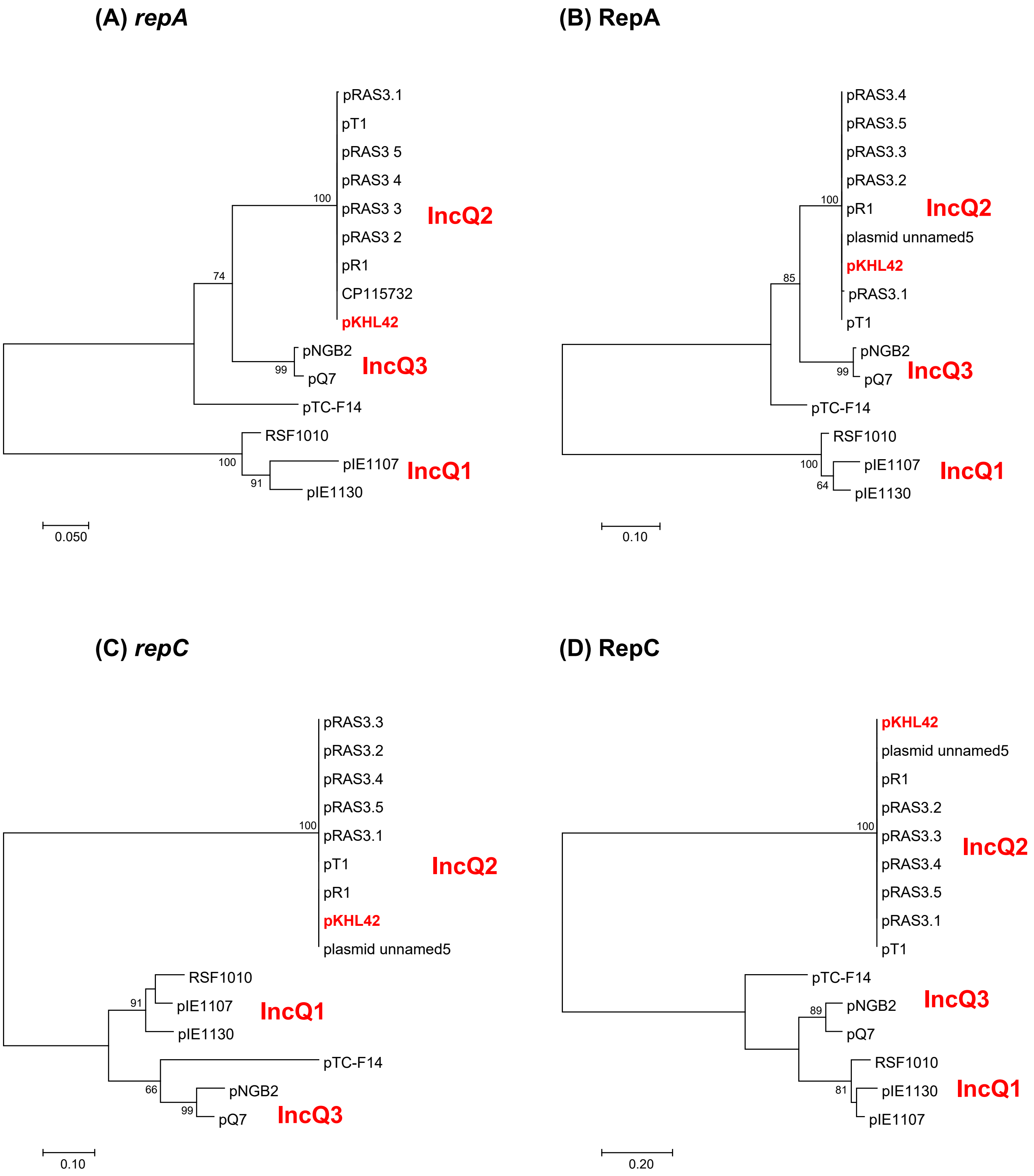

Figure S2. Phylogenetic trees of IncQ plasmids, inferred from nucleotide sequence data of 15 *repA* genes (A) and *repC* genes (B), and their amino acid sequence data of RepA proteins (C) and Rep proteins (D). Plasmids obtained in this study are shown in red. Bootstrap percentages at nodes were calculated with 1000 replicates. The tree is drawn to scale, with branch lengths measured in the number of substitutions per site (indicated by horizontal scale bars). Accession numbers of nucleotide sequences are shown in Table S2.

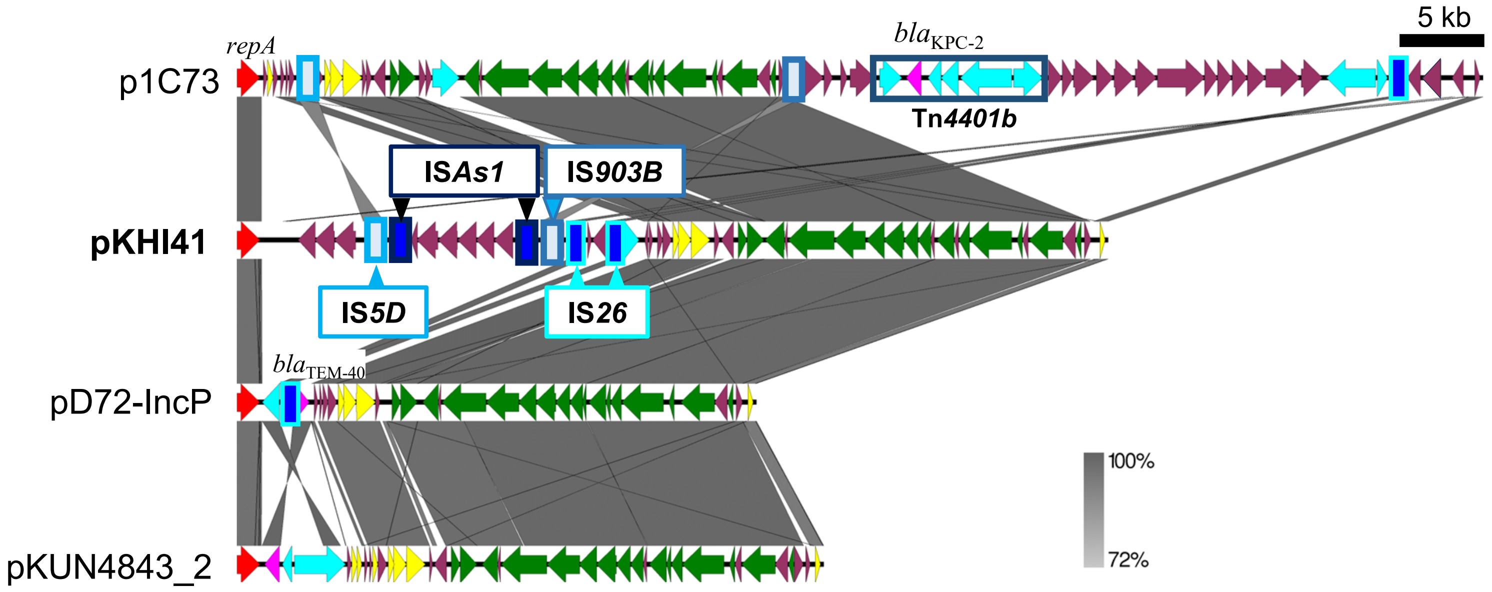

Figure S3. Alignments of unclassified plasmids with rep_cluster_312 RIP gene isolated from different WWTPs. Coding DNA regions, their directions, and their predicted functions are indicated as block arrows with colors, red for replication, green for conjugation, yellow for other genes in each backbone (shown above or below each plasmid), light blue for genes related to mobile genetic element, pink for genes related to antimicrobial resistance genes, and magenta for other accessory genes. Accession numbers of nucleotide sequences of similar plasmids are shown in Table S2.
